# Comparison of apical and basolateral Cu treatment for iron-related gene regulation during deferoxamine induced iron deficiency

**DOI:** 10.1186/s12263-022-00717-8

**Published:** 2022-12-09

**Authors:** Ezgi Evcan, Sukru Gulec

**Affiliations:** İzmir Institute of Technology, Faculty of Engineering, Department of Food Engineering, Molecular Nutrition and Human Physiology Laboratory, Urla, 35430 İzmir, Turkey

**Keywords:** Iron deficiency, Anemia, Copper, Iron, Intestine, Caco-2, Apical, Basolateral

## Abstract

**Background:**

Intestinal copper transporter (Atp7a) mutant-brindled mice with systemic Cu deficiency had elevated Cu levels in enterocyte cells without any perturbation of iron-regulating genes, suggesting that blood Cu level might be important for intestinal iron homeostasis during iron deficiency (ID). We hypothesized that the blood Cu level and polarization (apical and basolateral) of enterocyte cells might be important regulators for the compensatory response on the regulation of genes in enterocyte cells during iron deficiency.

**Methods:**

We grew Caco-2 cells on a bicameral cell culture plate to mimic the human intestine system and on a regular tissue culture plate. Iron deficiency was induced by deferoxamine (DFO). The cells were treated with Cu and Cu with Fe following mRNA expressions of *DMT1*, *FPN*, *TFR*, and *ANKRD37* were analyzed.

**Results:**

Our main finding was that basolateral treatment of Cu significantly reduced mRNA expressions of iron-regulated genes, including *DMT1*, *FPN*, *TFR*, and *ANKRD37*, compared to DFO-treated and DFO with apical Cu-treated groups in both bicameral and regular tissue culture plates.

**Conclusions:**

Cu level in the basolateral side of Caco-2 cells significantly influenced the intracellular gene regulation in DFO-induced iron-deficient condition, and polarization of the cells might be important factor gene regulation in enterocyte cells.

## Background

Iron is an essential and vital nutrient for life to sustain the functions of living organisms. In the human body, iron is essential to carry oxygen to tissues since it is the main component of hemoglobin. Moreover, it is necessary to control cell division and differentiation, mitochondrial energy production, DNA replication and repair, and immune response against pathogenic microorganisms [1, 2]. Iron levels in the human body are tightly controlled via intestinal absorption since mammalians do not have a specific active iron excretory mechanism. Therefore, intestinal enterocyte cells maintain adequate levels of iron in the body. Inadequate dietary iron absorption through enterocyte cells of the intestine leads to iron deficiency, one of the major global nutritional deficiencies, especially in women, infants, and children. Iron deficiency causes elevated copper mineral levels in the intestinal mucosa [3], liver, and serum [4] in many mammalian species, including humans [5]. Previous investigations have implicated that copper homeostasis perturbations influence iron metabolism [1, 5–8].

Divalent metal transporter (*DMT1*), the iron exporter ferroportin 1 (*FPN*1), can be a potential connection between iron and copper metabolisms since there are studies that suggest iron amount is regulated by copper in intestine [9–11]. Intestinal hypoxia-inducible factor 2α (HIF-2α) is essential for iron absorption during iron deficiency since it regulates apical and basolateral iron transporters [12]. Copper influences the DNA-binding activity of HIF-2α, illustrating another connection of copper influencing iron homeostasis. *DMT1* and *FPN* were shown as direct target genes related to HIF-2α [13]. mRNA regulation of Ankyrin repeat domain 37 (*ANKRD37*), prolyl 4-hydroxylase (*P4ha1*), and HIF prolyl hydroxylase 3 (*EGLN3*) are the most well-known marker genes for the hypoxic signal under iron deficiency.

Recent studies have shown that the regulation of genes involved in iron and copper homeostasis were altered in brindled mice [9]. However, when intracellular Cu level increased during iron deficiency, Cu did not affect the genes’ regulation, which plays a role in enterocyte iron metabolism. In the same study, enterocyte cells of the mice intestine were treated with high intracellular and low-blood Cu levels. Thus, blood Cu level might be a more dominant regulator of iron metabolism-related genes during iron deficiency. The apical side of the enterocyte is in contact with dietary nutrients, whereas the basolateral part is in contact with the blood. Considering this apical and basolateral polarization, different sides of enterocytes which in relation to different environmental conditions (diet or blood) may be necessary for controlling the molecular and genetic regulation of iron metabolism in enterocyte cells. However, to the best of our knowledge, no research exists related to the dependency of the apical versus basolateral Cu for enterocyte cell iron metabolism during iron deficiency. Given this background, the current study investigated the effects of dietary and blood copper treatments separately on iron and iron-dependent hypoxic gene regulations of anemic enterocyte cells by in vitro modeling of the human intestine system.

## Results

### Modeling of human intestine system and TEER measurements of monolayer Caco-2 cells

**Fig. 1:**
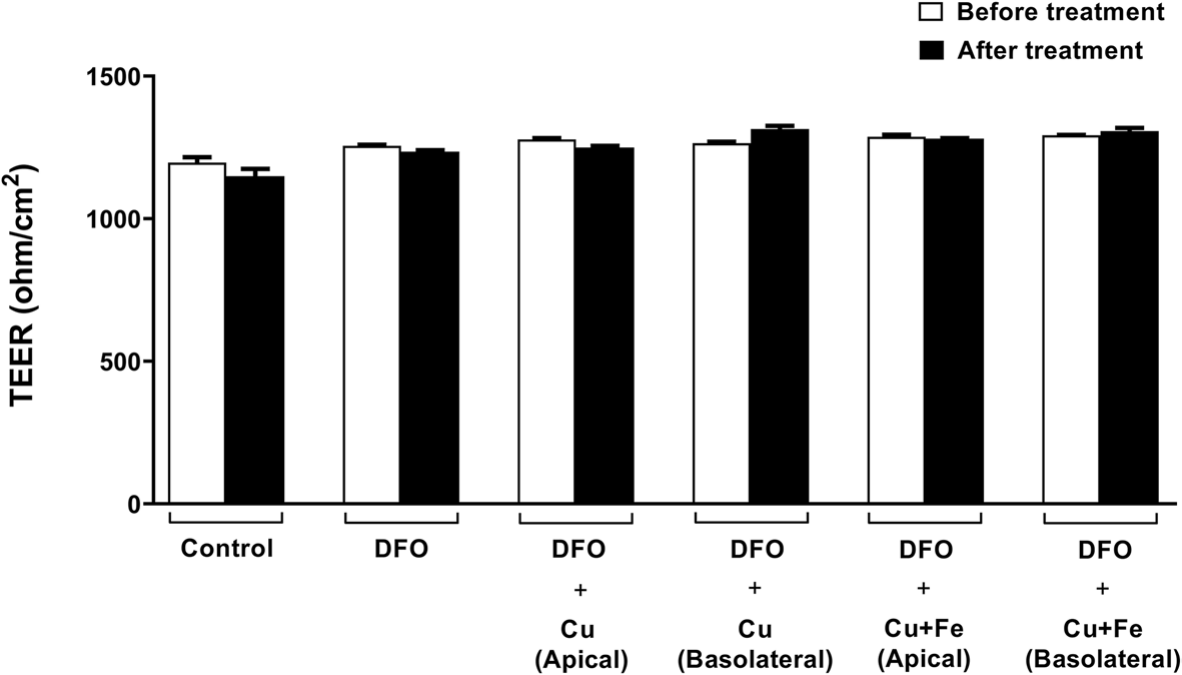
Transepithelial electrical resistance (TEER) measurements. The Caco-2 cells were grown on the bicameral insert for 21 days. TEER measurements controlled monolayer cell polarization and tight junction integrity of the Caco-2 cells

The apical and basolateral polarization of Caco-2 cells can behave like the human small intestine [14]. Therefore, Caco-2 cells were grown on the bicameral cell culture system for 21 days and then DFO and mineral treatments were performed. TEER was measured from all experimental groups at the end of 21 days and after treatments to control the stability of the monolayer integrity of the cells. As shown in the Fig. 1, we did not observe any significant change in TEER values between the experimental groups before and after Cu and Fe treatments. Furthermore, TEER values that are much higher than 250 ohm/cm^2^ reflect the experimentally polarized cells (as apical and basolateral) in the human intestinal system.

### Apical and basolateral treatments of Cu in DFO-induced anemic monolayer Caco-2 cells

**Fig. 2:**
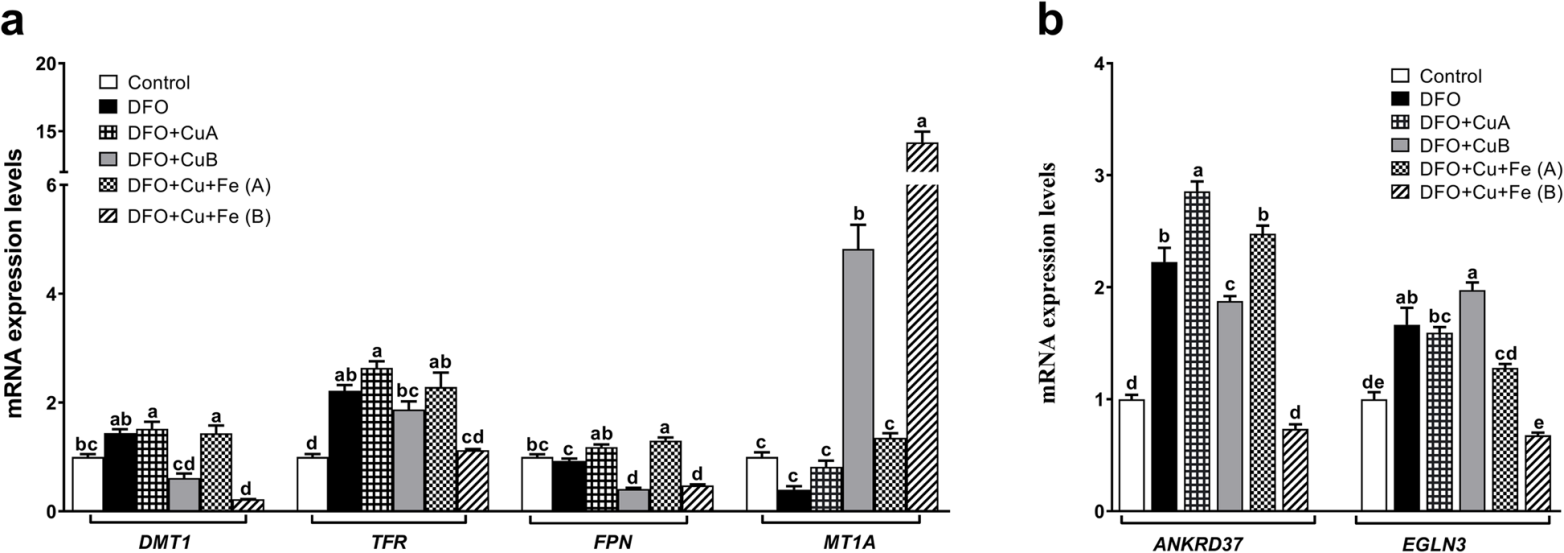
The effect of trace minerals on iron and hypoxia-regulated gene mRNA expression levels in polarized Caco-2 cells under the DFO treatment condition. Caco-2 cells were grown on the bicameral cell culture system for 21 days. Iron deficiency was induced by 200 μM for 24h, and then, the copper (CuCl_2_; 100 μM) and iron (FAC; 100 μg/mL) were given to apical and basolateral sides of the cell culture system for 18h. The effects of Cu and Cu with Fe on iron (**a**) and hypoxia (**b**) regulating gene mRNA expression were analyzed

When the Caco-2 cells are grown on the bicameral cell culture system, it allows us to study the effects of apical and basolateral Cu treatment on regulation of iron metabolism-related genes in enterocyte cells during DFO-induced iron deficiency. DFO treatment induced *DMT1*, *TFR*, and *ANKRD37*, and *EGLN3* mRNA levels; however, *FPN* and *MT1a* were not affected (Fig. 2a). When Cu was given to the apical side of the cells, it did not affect mRNA levels of genes, including *DMT1*, *TFR*, and *ANKRD37*, and *EGLN3*, that were induced by DFO. Cu treatment of the basolateral side of cells significantly reduced *DMT1*, *FPN*, and *TFR*, and *ANKRD37* mRNA levels under the iron deficiency condition. Moreover, *DMT1*, *FPN*, and *TFR* gene expressions were not changed by apical side treatments of the cells by Cu and Fe, whereas *ANKRD37* and *EGLN3* mRNA levels were slightly reduced. Furthermore, when both Cu and Fe were given to the basolateral side of the cells, mRNA levels of iron regulating genes (*DMT1*, *FPN*, and *TFR*) and hypoxia-related genes (*ANKRD37* and *EGLN3*) were significantly lower than all treatment groups. *MT1a* mRNA levels were significantly increased by basolateral Cu treatment of cells, and this induction was higher when both Cu and Fe were given to the basolateral side of the cells (Fig. 2a, b).

### The effect of Cu on iron deficiency in Caco-2 cells grown on the 12-well tissue culture plate

**Fig. 3:**
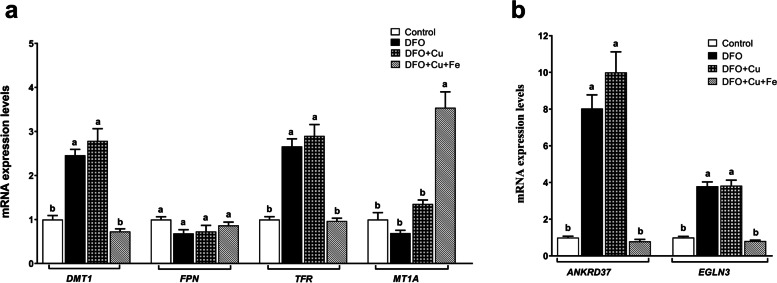
The effect of minerals on iron-regulated gene mRNA expression levels in Caco-2 cells under the DFO treatment condition. Caco-2 cells were grown on the 12-well tissue culture plates for 21 days. The cells were treated by 200 μM for 24h to induce iron deficiency. The copper mineral (CuCl_2_; 100 μM) and iron (FAC; 100 μg/mL) were given to Caco-2 cells for 18h to test the effect of Cu and Cu with Fe on iron- (**a**) and hypoxia- (**b**)regulated genes mRNA expressions

Copper and iron interaction in enterocyte cells has attracted interest since their homeostasis is controlled by intestinal absorption. In this study, we grew Caco-2 cells on 12-well tissue culture plates for 21 days and then treated by DFO to induce iron deficiency. We treated the cells Cu with and without Fe. Then, we analyzed the mRNA levels of iron regulating and iron-dependent hypoxia regulating genes to see the effects of Cu on iron metabolism during iron deficiency (Fig. 3a, b. DFO treatment significantly induced *DMT1*, *TFR*, and *ANKRD37*, and *EGLN3* mRNA expression levels, whereas *FPN* and *MT1a* mRNA levels were not affected. The Cu treatment did not reduce DFO-induced gene mRNA expressions and did not affect *FPN* and *MT1a* mRNA levels. However, Cu with Fe treatment significantly reduced *DMT1*, *TFR*, *ANKRD37*, and *EGLN3* mRNA levels. Moreover, we observed that Cu and Fe together significantly upregulated *MT1a* mRNA expression.

## Discussion

Enterocyte cells are unique cell types in the intestine due to the interaction between diet and the internal circulatory system of the body. Thus, the dietary and blood-derived stimulus might play a role in enterocyte nutrient metabolism. Copper and iron have physiological interactions in enterocyte cells during iron deficiency. In many mammalian species, iron deficiency leads to elevated Cu levels in enterocyte [15], serum [16], and liver [17], indicating that copper might influence intestinal iron metabolism. Iron and copper interactions have been investigated in brindled mice (Mo^*Br/y*^) and researchers observed no perturbation in iron absorption during anemia [9]. In this study, the enterocyte copper level of mutant mice was high due to nonfunctional ATP7A protein. In contrast, these mice were systemic copper-deficient; thus, enterocyte cells were exposed to two different copper levels. Furthermore, it was observed that dietary-induced iron deficiency upregulated mRNA expressions of iron-regulated genes, including *DMT1*, *TFR*, and *ATP7A*, but high enterocyte copper level did not affect the gene regulations suggesting that blood Cu level might be an important dietary factor for gene regulations in enterocyte cells during iron deficiency. Thus, we grew Caco-2 cells on a bicameral cell culture plate to mimic the human intestine system to test whether dietary (apical side) or blood copper (basolateral side) are involved in iron-dependent genes regulation during iron deficiency.

In the current study, we selected marker genes that were regulated by iron deficiency including iron transporters (*DMT1*, *TFR*, and *FPN*) and iron-dependent hypoxic genes (*ANKRD37* and *EGLN3*). Furthermore, *MT1A* mRNA level was used to control intracellular mineral uptake since *MT1A* is regulated by intracellular Cu and Zn, respectively [18, 19]. Our results showed that Cu and Cu with Fe in the basolateral side of the cells reduced marker genes mRNA levels (*DMT1*, *FPN*, *TFR1*, and *ANKR37*) compared to the apical Cu and Cu with the Fe treatment group during iron deficiency. This suggests that Cu sensing of the basolateral side of the cells might be different from Cu sensing of the apical side. Cu regulates the *MT1A* mRNA levels, and its level is correlated by elevated intracellular Cu [19]. We observed that *MT1A* mRNA levels were significantly induced when Cu and Cu with Fe were given to the basolateral side of the cells suggesting that the level of Cu in the blood might be a compelling factor for the regulation of iron deficiency-related genes in the intestine. Furthermore, the basolateral side of the enterocyte cells may play a role in Cu sensing. The importance of the molecular and genetic interactions between basolateral and apical sides of polarized enterocyte cells has been indicated in different studies. It was shown that glucose treatment in the basolateral side of the Caco-2 cells induced apical cholesterol uptake and the mRNA levels of the cholesterol transporter gene [20], indicating that basolateral signaling influences fatty acid metabolism through apical side of enterocyte cells*.* Moreover, Han et al. (2002) [21] showed that both apical and basolateral Cu treatment increased Fe uptake in non-anemic Caco-2 cells. Moreover, Cu treatment of apical and basolateral sides also induced *DMT1*, *TFR*, and *FPN* mRNA levels compared to the control group. All together results suggest that basolateral and apical sides of enterocyte cells might have a different physiological response regarding the same nutritional stimulus.

Caco-2 cells can differentiate on the regular tissue culture plate when they are grown 21 post-confluent days. Differentiation of Caco-2 cells gives different genetic responses for a variety of cellular physiological pathways [14]. However, polarization of those cells on the insert system might be another factor that can influence genetic regulation. Thus, we also grew Caco-2 cells on regular tissue culture plates at the same time that we performed bicameral cell culture experiments. We observed similar results for marker genes regulation between apical side Cu treatment groups and Cu treatment on Caco-2 cells that were grown on tissue culture plates. However, basolateral Cu treatment affected regulations of *DMT1*, *FPN*, *MT1A*, and *ANKRD37* genes compared to results from the cells that were grown on tissue culture plates. Our results suggest that the polarization of the Caco-2 cells might be an important factor for gene regulation in terms of Cu treatment in our study. It might be better to account polarization of the Caco-2 cells for gene regulation or whole-genome array studies.

## Conclusion

The nutrient-dependent regulation of enterocyte cells is central to the intestinal nutrient-sensing mechanism. The basolateral and apical sides of the enterocyte cells are the primary targets to understand nutrient sensing in terms of nutrient overload or deficiency. Furthermore, polarization of Caco-2 cells also might influence gene regulation. Our results suggest that intracellular gene regulation was mainly affected by Cu treatment in the basolateral side of enterocyte cells during iron deficiency, indicating that blood copper level might have ability to control the enterocyte iron metabolism at molecular and genetic levels during iron deficiency. The blood Cu level might be an important regulator for intestinal iron metabolism during iron deficiency.

## Methods

### Cell culture

The human colorectal adenocarcinoma epithelial cell line, Caco-2, was purchased from the American Type Culture Collection (ATCC, HTB-37, Manassas, VA). Caco-2 cells were cultured in minimal essential medium (MEM) (Sigma, United Kingdom) supplemented with 20% fetal bovine serum (FBS) (Gibco, Cat. No. 10500), 1% penicillin and streptomycin (100 U/mL), and 1% nonessential amino acid solution (Gibco, Cat. No. 11140). They were maintained in 75 cm^2^ culture flasks at 37°C in a constant humidified incubator with an air atmosphere of 5% CO_2_/95% O_2_. When cultures reached 70–80% confluency, they were plated for either subsequent passage or treatment. Caco-2 cells used for the treatment experiments were between the 20th and 30th passages.

### Modeling of the human intestinal system and Cu treatments of apical and basolateral sides of the cells during iron deficiency

To test the effect of the dietary and blood copper on iron deficiency, we mimicked the human intestines. The Caco-2 cells were seeded into bicameral collagen-coated polytetrafluoroethylene membrane with 0.4-μm pore size and 1.12 cm^2^ diameter (12-well inserts) (Corning, Cat. No.: 3493). After 3 days of confluent culture, the cells were grown for an additional 21 days to form a monolayer and became polarized under standard conditions in the indicated MEM media. The experimental cell medium was changed every other day. Transepithelial electrical barrier resistance (TEER) was measured by an epithelial volt-ohm meter (EVOM; World Precision Instruments, Inc., FL, USA) to confirm the cell tight junction integrity. We only used cell monolayers with the TEER values above 250 Ω/cm^2^ for the treatment experiments (Sambuy et al. 2005). Iron deficiency was induced in the cells at 21-day of post-seeding using a chemical agent (deferoxamine, DFO) at a concentration of 200 μM. After incubation for 24 h, the apical and/or basolateral compartments of anemic Caco-2 cells were treated with the copper (copper (II) chloride, CuCl_2_; 100 μM) or iron (ferric ammonium citrate, FAC; 100 μg/mL) and/or together at the same time for further 18h in the incubator.

### Determination of the effect of copper on iron metabolism at the molecular and genetic level

The Caco-2 cells were seeded at 1×10^5^ cells/well into classical cell culture plates (12-well plates) (Costar, Cambridge, MA) to investigate the effects of Cu on molecular and genetic regulation of iron metabolism. Iron deficiency was induced in the cells at 21-day of post-seeding 200-μM DFO. After incubation for 24 h, anemic cells were treated with the copper and/or iron minerals at the same concentrations as previously described in the method section.

### Quantitative real-time polymerase chain reaction (qRT-PCR)

We evaluated the mRNA expression levels of the marker genes that regulate iron metabolism and hypoxic condition. The total RNA was isolated from cells with RNAzol reagent (MRC, Cat. No.: RN190) following the manufacturer’s protocol. One microgram of each total RNA was converted to cDNA using the cDNA synthesis kit (Lifetech, Cat. No.: 4368814). qRT-PCR was performed on an ABI StepOnePlus instrument (Lifetech, CA, USA) by using gene-specific oligonucleotide primers (*CYPA*: Forward-TACGGGTCCTGGCATCTTG, Reverse-CGAGTTGTCCACAGTCAGCA; *DMT1* Forward-TGCATCTTGCTGAAGTATGTCACC, Reverse-CTCCACCATCAGCCACAGGAT; FPN: Forward-GCAGGAGAAGACAGAAGCAAACT, Reverse-TCCTTCGAATTGTGGCATTCAT; *MT1A*: Forward-ACTGGTGGCTCCTGCACCTGCACT, Reverse-ACAGCAGCTGCACTTCTCTGAT; TFR: Forward-TCAGAGCGTCGGGATGATATCGG, Reverse-CTTGATCCATCATCATTCTGAACTGCC; *ANKRD37*: Forward AGCAGTCGCCTGTCCACTTAGC, Reverse-AGCAGGCTTAGGCACTCCAGG; *EGLN3*: Forward-GCAAATACTACGTCAAGGAGAGGTCTAA, Reverse-GGCATCCCAATTCTTGTTCAGATAG) SYBR-Green mix (Lifetech, Cat. No.: 4367659). C_T_ (threshold cycle) levels were normalized according to *CYPA* mRNA expression as a housekeeping gene. Mean fold changes in gene-specific mRNA levels from all experimental groups were calculated by the ^2-ΔΔCt^ analysis method [22].

### Statistical analyses

The results were expressed as the mean values ± standard deviation of three independent experiments with at least two parallels of each experimental group. Statistical analysis for more than two groups was carried out by one-way analysis of variance (ANOVA) with Tukey’s post hoc test by using GraphPad Prism 6 (GraphPad Software Inc., CA, USA). Data were considered significant for *p* ≤ 0.05.

## Data Availability

The datasets used and/or analyzed during the current study are available from the corresponding author on reasonable request.

## Abbreviations

Atp7a: Intestinal copper transporter
Caco-2: Human colorectal adenocarcinoma epithelial cell line
DFO: Deferoxamine
DMT1: Divalent metal transporter
FPN1: Ferroportin
TFR: Transferrin receptor
ANKRD37: Ankyrin repeat domain 37
HIF-2α: Intestinal hypoxia-inducible factor
P4ha1: Prolyl 4-hydroxylase
EGLN3: HIF prolyl hydroxylase 3
MT1a: Metallothionein-1A
CYPA: Cyclophilin A
ATCC: American Type Culture Collection
MEM: Minimal essential medium
FBS: Fetal bovine serum
TEER: Transepithelial electrical barrier resistance
EVOM: Epithelial volt-ohm meter
FAC: Ferric ammonium citrate
qRT-PCR: Quantitative real-time polymerase chain reaction
CT: Threshold cycle
